# Twenty-Year Course of Antifungal Resistance in *Candida albicans* in Türkiye: A Systematic Review and Meta-Analysis

**DOI:** 10.3390/jof11080603

**Published:** 2025-08-19

**Authors:** Imdat Kilbas, Elmas Pinar Kahraman Kilbas, Florin George Horhat, Ihsan Hakki Ciftci

**Affiliations:** 1 Medical Microbiology Doctorate Program, Institute of Health Sciences, Istanbul University, 34093 Istanbul, Türkiye; imdtklbs@gmail.com; 2 Department of Medical Laboratory Techniques, Health Services Vocational School, Fenerbahce University, 34758 Istanbul, Türkiye; elmspnrkk@gmail.com; 3 Department of Microbiology, Victor Babes University of Medicine and Pharmacy, Eftimie Murgu Square No. 2, 300041 Timisoara, Romania; 4 Department of Medical Microbiology, Faculty of Medicine, Sakarya University, 54100 Sakarya, Türkiye

**Keywords:** *Candida albicans*, antifungal resistance, systematic review, meta-analysis, Türkiye

## Abstract

This study aimed to systematically evaluate the resistance rates of *Candida albicans* to various antifungals based on studies conducted in Türkiye and published between 2005 and 2025 and to analyze the factors contributing to resistance. A systematic literature search was conducted using various keywords in electronic databases (PubMed, Embase, Web of Science, EBSCO, Scopus, Turk Medline and Google Scholar). A total of 42 studies were included in the meta-analysis according to the determined criteria. The quality of the studies was assessed using the Joanna Briggs Institute checklist, and the analyses were performed using appropriate statistical software. The highest resistance rates for fluconazole, itraconazole, and voriconazole were observed in the Aegean and Marmara regions. In the analyses performed with the random-effects model, heterogeneity was found to be high for itraconazole, fluconazole, posaconazole, voriconazole, and caspofungin, and the strongest explanatory variable of this heterogeneity was the geographical region variable. In our study, we determined that antifungal resistance in *C. albicans* strains in Türkiye is generally low; however, an increasing trend has been observed over the years, especially in amphotericin B resistance. Although the low resistance rates to major antifungal agents such as fluconazole, voriconazole and echinocandins are promising, regional differences and methodological heterogeneity necessitate the development of treatment strategies based on local data.

## 1. Introduction

*Candida albicans* is a member of the natural human microbiota and colonizes the gastrointestinal and reproductive tracts, oral cavity, and skin of most humans as a commensal organism [1]. The failure to identify a possible environmental reservoir for *C. albicans* suggests that this species is perfectly adapted to healthy mammalian hosts. However, benign commensal colonization can become pathogenic due to changes in pH and oxygen levels, host microbiota, or host immune response [2]. Over the past two decades, the prevalence of non-albicans *Candida* species has surpassed *Candida albicans* infections in many geographic regions worldwide, highlighting the need for comprehensive measures to develop effective treatment strategies and prevent future outbreaks. A meta-analysis from Iran determined that non-*albicans Candida* species were responsible for 72.7% of all candidemia cases. *Candida parapsilosis* was the most frequently isolated species (30.8%), followed by *C. albicans* (27.3%), *Candida glabrata* (18.2%), and *Candida tropicalis* (14.5%) [3]. Another study identified *C. albicans* (50%), *Candida krusei* (20%), *Candida glabrata* (10%), *C. tropicalis* (10%), and *C. parapsilosis*, respectively [4]. *Candida parapsilosis* is the second or third most frequently isolated species from patients and is one of the most common causes of candidemia worldwide. *Candida parapsilosis* accounts for more than 20% of *Candida* species isolated from blood cultures in Brazil, Argentina, Peru, Spain, Russia, and China, and more than 30% in Türkiye, Greece, Croatia, Romania, South Africa, Nigeria, and Paraguay [5].

However, *C. albicans* is important in the development of superficial and systemic fungal infections [6]. The increased number of *Candida* infections has been associated with conditions such as excessive use of broad-spectrum antifungals and antibiotics, chemotherapy, organ transplantation, the presence of indwelling catheters, and poor performance of routine microbiological tests [2]. In 2012, the annual incidence of invasive candidiasis was estimated to be approximately 400,000 cases globally [7]. The increase in this rate was remarkable. Reports for 2024 stated that approximately 1.5 million people developed invasive candidiasis each year and approximately 1 million people died due to it (63.6%) [8].

Antimicrobial resistance is a major public health problem worldwide and a concern, especially for fungal infections. Increased mortality rates are associated with the limited number of antifungal drugs to treat invasive fungal infections and the prevalence of multidrug-resistant (MDR) fungal pathogens [9]. Since the 1980s, azoles, especially fluconazole, which are effective orally, have emerged as an important tool for treating systemic fungal infections without the nephrotoxicity associated with amphotericin B treatment. However, the current use of antifungal agents raises concerns regarding their potential to select and spread resistant fungal strains or species [10].

*Candida albicans* has become a serious global health problem due to its ability to develop resistance to antifungal agents. Resistance to commonly used antifungal drugs, especially azoles (fluconazole and voriconazole), echinocandins (caspofungin and micafungin), and polyenes (amphotericin B), complicates the treatment of infections. In Türkiye, the average resistance rates of *C. albicans* against azole antifungals were reported as 9.6 ± 17.8% for fluconazole, 14.6 ± 26.3% for voriconazole, 23.2 ± 33.1% for itraconazole, 8.6 ± 9.5% for micafungin from the echinocandin group, and 2.5 ± 5.8% for amphotericin B from polyenes, and this resistance was reported to be increasing over time [11]. Türkiye, situated between Europe and Asia, is a country that hosts a large number of immigrants, boasts a strong health tourism scene, and has extensive antimicrobial use. Therefore, evaluating antifungal resistance data from Türkiye will contribute to the literature by providing an understanding of national and global resistance trends. This study aimed to systematically assess studies reporting *C. albicans* resistance rates to various antifungal drugs in Türkiye between 2005 and 2025, compare diagnostic methods, and analyze resistance trends over time.

## 2. Material and Methods

### 2.1. Protocol

The current study was designed in accordance with the PRISMA guidelines for systematic reviews and meta-analyses [12] and registered in the PROSPERO database under the number CRD420251060431.

### 2.2. Literature Search

A comprehensive systematic search was conducted in English and Turkish in the electronic databases PubMed, Embase, Web of Science, EBSCO, Scopus, Turk Medline, and Google Scholar between January 2005 and January 2025. The keywords “*Candida* türlerinin antifungal direnç/duyarlılık durumu,” “Türkiye/Turkey,” “*Candida albicans* antifungal direnç/duyarlık durumu,” “antifungal duyarlılık,” “*Candida* species antifungal resistance/susceptibility,” “*Candida albicans* antifungal resistance/susceptibility,” and “antifungal susceptibility” were used for the search.

Four independent researchers determined the keywords to be used in the literature search and assessed them for appropriateness. The titles and abstracts of the retrieved studies were independently screened according to the predefined inclusion and exclusion criteria. The full texts of the eligible studies were then examined in detail, and research articles that met all inclusion criteria were included in the study. Rayyan-Qatar Computing Research Institute software (https://www.rayyan.ai/about-us, Accessed date: 2 June 2025) was used to manage the records obtained from the literature search. Duplicate records were eliminated automatically, and the title-abstract level was assessed preliminarily for eligibility using this software.

### 2.3. Inclusion Criteria

The study included research articles published between 2005 and 2025, with accessible full text, describing species at the species level, containing at least 20 strains, published in Turkish or English, and published in national or international peer-reviewed journals.

### 2.4. Exclusion Criteria

Studies that did not provide species-level definitions, could not be accessed in full text, did not report species-level resistance/susceptibility rates, were conducted with fewer than 20 strains, were conducted with fewer than two antifungals, had inconsistent data, studied different numbers of strains for each antifungal, were conducted with strains isolated from animals and the environment, and were published before 2005, and articles written in languages other than Turkish and English, compilations, systematic reviews, meta-analyses, case reports, congress proceedings, and book chapters were not included in the meta-analysis.

### 2.5. Quality Control of Data

The authors assessed the quality of the studies using the Joanna Briggs Institute’s prevalence studies checklist. This list included nine questions answered by the raters for each of the studies. A “Yes” answer was scored as 1 point. The total score of each study varied between 0 and 9. Studies with scores between 4 and 6 were considered to be of medium quality, and those with scores between 7 and 9 were considered to be of high quality. Research articles with scores lower than four were excluded, and articles with scores between 4 and 9 were included in the meta-analysis [13].

### 2.6. Data Analysis

During the literature review, two authors (I.K. and E.P.K.K) reviewed the articles’ titles and abstracts independently, and then the full texts were obtained. Microsoft Excel spreadsheets were used to collect the data. This table lists the first author’s last name, publication year, place of the study, sample size, identification methods used, and antifungal resistance detection methods used. Data were analyzed using SPSS (IBM SPSS Statistics, Version 25.0; IBM Corp., Armonk, NY, USA). One-way analysis of variance (ANOVA) evaluated whether the antifungal resistance rate differed significantly over time. The significance level was set at *p* < 0.05. The significance of the difference between the groups was examined using Tukey’s test, a post hoc analysis. Heterogeneity between the studies included in the meta-analysis was assessed through forest plots and the I^2^ heterogeneity coefficient using CMA (Version 3.0, Biostat, NJ, USA). A fixed or random effects model was preferred according to the obtained test results. Forest plots were created to visually summarize individual study results and the overall effect size. 95% confidence intervals (CIs) were calculated to demonstrate the statistical reliability of antifungal resistance rates reported in the included studies.

## 3. Results

### 3.1. Characteristics of the Studies

**Figure 1:**
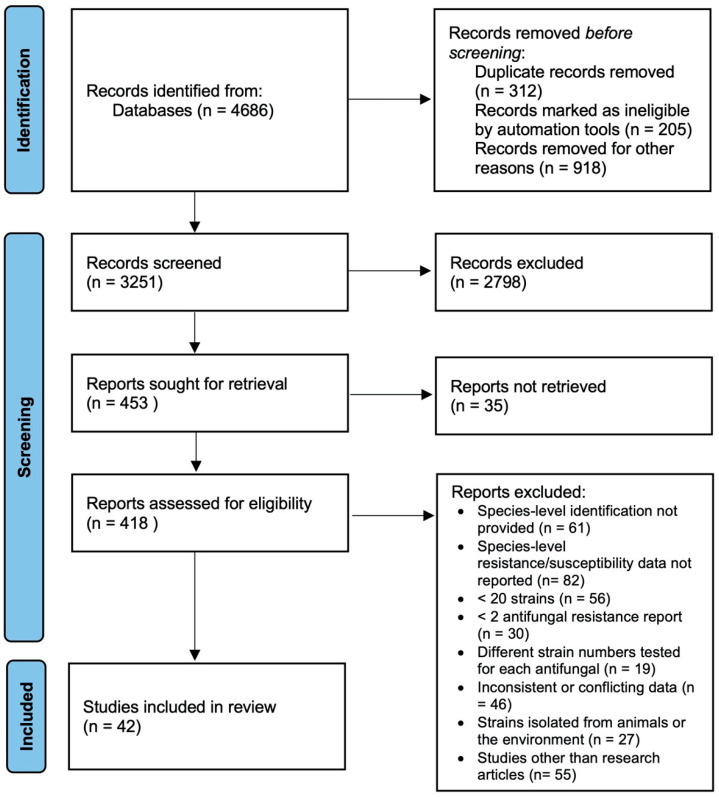
PRISMA flowchart of meta analysis.

The search conducted with the determined keywords in the databases identified a total of 4686 studies. A total of 1435 records were previously eliminated; 312 were duplicates, 205 were found inappropriate by automation, and 918 were excluded for other reasons. A total of 3251 records were assessed, of which 2798 were eliminated. A total of 418 full texts were evaluated, 376 were excluded, and 42 studies were included in the meta-analysis [14,15,16,17,18,19,20,21,22,23,24,25,26,27,28,29,30,31,32,33,34,35,36,37,38,39,40,41,42,43,44,45,46,47,48,49,50,51,52,53,54,55] (Figure 1).

The studies were examined in seven groups according to the geographical regions of Türkiye: Eastern Anatolia, Black Sea, Southeastern Anatolia, Central Anatolia, Marmara, Mediterranean, and Aegean regions. Table 1 summarizes the characteristics of all the included studies.

According to the quality assessment of the studies, 17 (40.47%) were of medium quality, and 25 (59.53%) were of high quality. The included studies were analyzed in three periods: 2005–2014 (*n* = 13), 2015–2018 (*n* = 15), and 2019–2025 (*n* = 14).

2015 marked a significant change in the standards for evaluating antifungal susceptibility testing in Türkiye [56]. During this period, in addition to the long-used Clinical Laboratory Standards Institute (CLSI) criteria, the European Committee on Antimicrobial Susceptibility Testing (EUCAST) guidelines began to be implemented in laboratory practice in our country. Because there were differences in the methods, breakpoints, and interpretation criteria used between the CLSI and EUCAST guidelines, changes were observed in resistance profiles. The period 2015–2018 described in the article represents an adaptation period during which this methodological transition and laboratory harmonization took place.

In 2019, EUCAST implemented significant terminology and definition changes regarding the interpretation of antifungal susceptibility testing. Since this date, the definitions of susceptibility categories S, I, and R have been revised [57]. These changes are structured to strengthen the relationship between clinical dosing strategies and pharmacokinetic/pharmacodynamic principles. This revision has led to differences in the resistance classifications of antifungal agents compared to previous years. The period 2019–2025 represents a period in which this new classification approach was established and integrated into clinical practice and was evaluated as a separate period in the analyses due to the potential for methodological incompatibility with previous years.

VITEK 2 was the most commonly used identification method in the studies (18/42), whereas API ID 32C (*n* = 14), API ID 20C AUX (*n* = 7), and MALDI-TOF MS (*n* = 5) were the most frequently used methods. The most commonly used method to determine antifungal susceptibility was VITEK 2, which was used in 17 studies (40.47%). This was followed by broth microdilution in 10 studies (23.80%) and E-test methods in 9 studies (21.42%). Of the studies, 57.14% used the Clinical and Laboratory Standards Institute (CLSI) as a guide, 16.66% used the European Committee on Antimicrobial Susceptibility Testing (EUCAST), and 4.76% used both guides together. However, 21.44% of the studies did not state the guide used. Blood (*n* = 32) was the most frequently studied clinical sample, followed by urine (*n* = 17), wound swabs (*n* = 10), and sputum (*n* = 9) (Table 1).

All studies included a total of 5569 *C. albicans* strains. It was determined that the strains were resistant to fluconazole by an average of 4.45% (*n* = 248), voriconazole by 4.37% (*n* = 181), itraconazole by 4.42% (*n* = 128), amphotericin B by 2.10% (*n* = 117), caspofungin by 2.94% (*n* = 70), micafungin by 1.95% (*n* = 49), posaconazole by 2.91% (*n* = 45), flucytosine-5 by 0.29% (*n* = 16), and anidulafungin by 1.05% (*n* = 6) (Table 2). Only one article studied nystatin, reporting the resistance rate to nystatin as 4.34% [14]. Two articles studied ketoconazole; one reported all strains as ketoconazole-susceptible, and the other reported 32% of strains as ketoconazole-resistant [14,15]. Our study found the highest resistance rate to amphotericin B in the Mediterranean region (4.82 ± 4.29%). Studies conducted with amphotericin B in the Eastern Anatolia region have shown that all strains are susceptible. The highest resistance rate to flucytosine-5 was found in the Black Sea region. On the contrary, all strains were susceptible to flucytosine-5 in the Southeastern Anatolia, Eastern Anatolia, Mediterranean, and Aegean regions. The highest resistance rates to fluconazole and voriconazole (13.97 ± 14.30% and 15.23 ± 8.21%, respectively) were found in the Aegean region. Itraconazole resistance reached its highest level in the Marmara region (23.91 ± 33.82%). The highest resistance rate to caspofungin was detected in the Mediterranean region (8.57 ± 10.86%); however, all strains were susceptible to caspofungin in studies in the Southeastern Anatolia, Mediterranean, and Black Sea regions. Considering the resistance rates of *C. albicans* to antifungal drugs by year, the highest resistance rates were determined as 4.60 ± 6.47, 3.44 ± 4.66, and 1.93 ± 3.21 for fluconazole, itraconazole, and voriconazole, respectively, between 2005 and 2014. The highest resistance rates were determined as 16.67 ± 19.05, 8.69 ± 13.81, and 7.51 ± 12.64 for itraconazole, fluconazole, and voriconazole, respectively, between 2015 and 2018. Between 2019 and 2025, itraconazole, fluconazole, and voriconazole resistance rates were 12.17 ± 14.67, 7.35 ± 9.93, and 6.70 ± 6.93, respectively. In general, flucytosine-5 (0.31 ± 0.85), caspofungin (1.99 ± 4.68), and amphotericin B (2.54 ± 3.37) were antifungals with the lowest resistance rates.

An increasing trend was observed for Amp B resistance over the years. While the resistance rate was 0.27% between 2005 and 2014, it increased to 4.50% between 2015 and 2018 and was 2.74% between 2019 and 2024. For 5-FC, the resistance rate was determined to be 0% between 2005 and 2014, 0.60% between 2015 and 2018, and 0.40% between 2019 and 2024. FLC resistance rates were 2.07%, 3.77%, and 3.90% in the 2005–2014, 2015–2018, and 2019–2024 periods, respectively, showing an increasing trend over the years. VRC resistance rates also increased similarly; they were found to be 0.33%, 3.28%, and 4.20%, respectively. A decrease was observed in ITZ resistance rates. The resistance rate, which was 2.18% between 2005 and 2014, decreased to 0.73% between 2015 and 2018 and was reported as 1.09% between 2019 and 2024. No resistant strains were detected for CAS and POS between 2005 and 2014. The resistance rate for CAS was reported to be 2.07% between 2015 and 2018, and no resistant strains were reported for POS. Data for the 2019–2024 period show a resistance rate of 1.93% for CAS and 0.62% for POS, indicating increased resistance to these agents over the years. No resistance data were reported for MFG and ANF between 2005 and 2014. All strains studied between 2015 and 2018 were found to be susceptible to these agents. However, between 2019 and 2024, a resistance rate of 1.79% was detected for MFG and 0.21% for ANF, and resistance rates were observed to increase over the years.

### 3.2. Meta-Analysis and Meta-Regression Analyses

**Figure 2:**
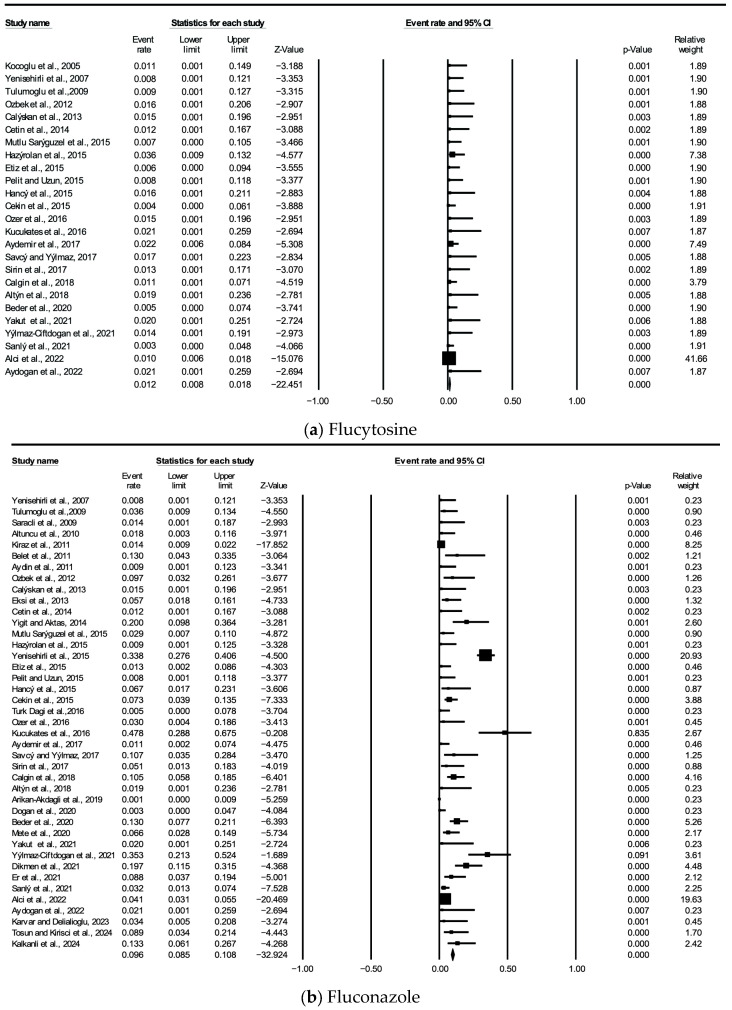

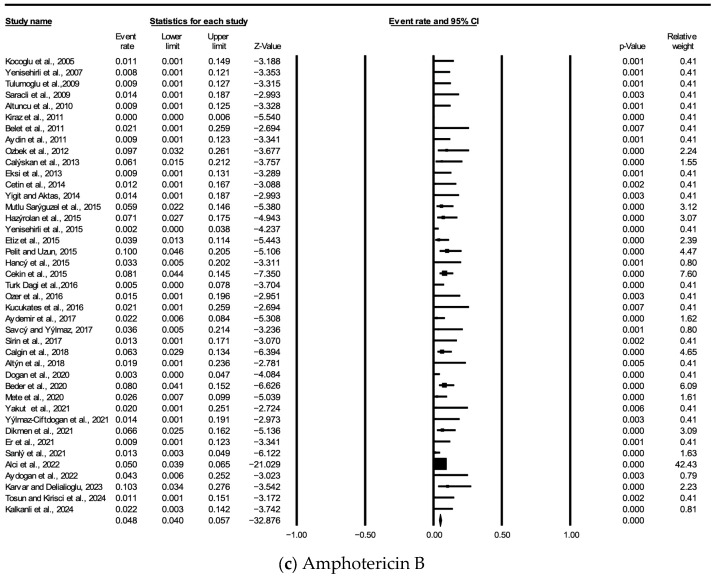
Forest plot graphs of the studies [14,15,16,17,18,19,20,21,22,23,24,25,26,27,28,29,30,31,32,33,34,35,36,37,38,39,40,41,42,43,44,45,46,47,48,49,50,51,52,53,54,55].

According to the random effects model, the mean effect sizes of the studies reporting resistance to micafungin (*n* = 12), anidulafungin (*n* = 8), amphotericin B (*n* = 41), and flucytosine (*n* = 25) were calculated as 0.028, 0.020, 0.038, and 0.012, respectively (*p* < 0.05). The heterogeneity levels were 43.57% for micafungin, 28.94% for anidulafungin, 34.67% for amphotericin B, and 0.0% for flucytosine, indicating a low level of heterogeneity. These results suggest that the studies were generally conducted using similar methodological approaches and the findings were consistent. According to the random effects model, the effect sizes were 0.071 for itraconazole (*n* = 17), 0.023 for caspofungin (*n* = 19), 0.035 for posaconazole (*n* = 9), 0.051 for fluconazole (*n* = 41), and 0.055 for voriconazole (*n* = 38), which were statistically significant (all *p* < 0.001). The heterogeneity levels were 90.89% for itraconazole, 88.73% for fluconazole, 78.07% for posaconazole, 73.35% for voriconazole, and 70.09% for caspofungin, all of which were evaluated as moderate to high [58]. For studies with high heterogeneity (itraconazole, caspofungin, posaconazole, fluconazole, and voriconazole), meta-regression analyses were conducted to identify the sources of heterogeneity. Resistance rates for each antifungal agent were summarized using forest plots. Confidence intervals were narrow, and the results were statistically significant (*p* < 0.05) in most studies (Figure 2).

The meta-regression analysis on itraconazole resistance found that the geographical region where the study was conducted significantly affected heterogeneity (*p* = 0.01). The R^2^ analog value of the model was 42%, and the region variable explained approximately half of the heterogeneity.

The meta-regression analysis on caspofungin resistance determined that the geographical region of the study significantly affected heterogeneity (*p* = 0.01). The R^2^ analog value of the model was 68%, with the region variable explaining more than half of the heterogeneity.

The meta-regression analysis on posaconazole resistance revealed that the geographical region where the study was conducted significantly impacted heterogeneity (*p* = 0.00). The R^2^ analog value of the model was 100%, and the region variable explained all of the heterogeneity.

The meta-regression analysis on fluconazole resistance found that the geographical region of the study significantly affected heterogeneity (*p* = 0.01). The R^2^ analog value of the model was 28%, with the region variable explaining approximately one-third of the heterogeneity. Additionally, the antifungal susceptibility detection method significantly contributed to the heterogeneity in fluconazole resistance, explaining 7% of heterogeneity (*p* = 0.03). The meta-regression analyses on voriconazole resistance found that the geographical region of the study significantly impacted heterogeneity (*p* = 0.00). The R^2^ analog value of the model was 9%. Furthermore, the antifungal susceptibility detection method significantly contributed to the heterogeneity in voriconazole resistance, explaining 56% of the heterogeneity (*p* = 0.00) (Appendix A).

## 4. Discussion

*Candida* species are the most common etiological cause of invasive mycotic diseases in immunocompromised individuals, those undergoing invasive clinical procedures, and those who have experienced major trauma requiring prolonged treatment in intensive care units [9]. *C. albicans* has been reported as a leading cause of healthcare-associated bloodstream infections in the United States, with a mortality rate of approximately 40% despite antifungal therapy [9,59]. *C. albicans* resides as a harmless commensal in the oral cavity or gastrointestinal tract of healthy individuals. However, it can spread to the bloodstream and colonize internal organs, causing life-threatening systemic infections in severely immunocompromised patients [60]. It may also increase in conditions such as diabetes, antibiotic use, hormonal changes, foreign body use, mucosal tissue damage, and prolonged hospitalization [61]. Due to the accelerating global increase in antifungal resistance, *C. albicans* ranks second in terms of public health risk on the pathogen list published by the World Health Organization (WHO) to increase resistance awareness [62]. It is crucial to monitor antifungal resistance and *C. albicans* infections and carefully select appropriate antifungals. Therefore, the current meta-analysis examined studies published between 2005 and 2025 on *C. albicans* resistance rates to various antifungal agents.

In addition to national resistance averages, this meta-analysis revealed notable variation in antifungal resistance rates across geographic regions and over time. Resistance to some antifungal agents, particularly azoles, has increased significantly over time. Regional differences were also observed, with some regions trending to higher resistance, possibly due to differences in antifungal use practices, infection control policies, and local epidemiology.

A meta-analysis study from Iran reported amphotericin B resistance to be 7.2% in *C. albicans* strains isolated from various clinical samples [6]. Another meta-analysis study conducted in 2022 reported amphotericin B resistance to be 5.37% in orally isolated *C. albicans* strains [63]. In their international study, Arendrup et al. (2023) stated that all *C. albicans* strains were susceptible to amphotericin B [64]. Different meta-analysis studies found amphotericin B resistance for *C. albicans* to range from 0% to 8.5%. These studies reported the lowest and highest rates in Nigeria and South Africa, respectively [65,66]. A study by Castanheira et al. (2017) found all 1310 *C. albicans* strains to be susceptible to amphotericin B [67]. In the research articles included in our study, amphotericin B resistance in *C. albicans* strains was 2.10% throughout Türkiye, and resistance increased over the years. While the studies by Arendrup et al. (2023) and Castanheira et al. (2017) reported that a large portion of *C. albicans* strains were susceptible to amphotericin B, it is noteworthy that this susceptibility started to decrease in our study [64,67]. Differences in amphotericin B resistance may arise from the sources of strains, test methods, guidelines used, and methodological differences in studies. The low resistance rate in Türkiye may be related to the limited use of amphotericin B and regional treatment policies. However, the increasing resistance trend over the years in this study can be explained by increased antifungal use, proliferation of biofilm-forming strains, and an increased number of immunosuppressed patients. Therefore, it is important to carefully monitor antifungal resistance trends and keep treatment approaches updated.

A meta-analysis study conducted on immunosuppressed patients between 2005 and 2015 reported fluconazole resistance of *C. albicans* strains to be 49.1%, 50%, 14.3%, 16%, 10.34%, and 0% for South Africa, Cameroon, Nigeria, Ethiopia, India, and Tanzania, respectively [66]. Additionally, some studies have reported that all strains are susceptible to fluconazole [67,68,69]. This meta-analysis detected fluconazole resistance in 4.45% of *C. albicans* strains, which is quite low and promising compared to the high resistance values in the literature. The low rates of fluconazole resistance detected in *C. albicans* strains in this meta-analysis indicate that this antifungal agent remains an effective treatment option in Türkiye. The high resistance rates detected in regions, such as Africa, can be explained by uncontrolled antifungal use, health infrastructure problems, and intensive antifungal exposure due to high HIV prevalence. The low resistance rate in Türkiye suggests that controlled drug use, effective infection control measures, and more selective antifungal use strategies can prevent the development of resistance. In particular, practices such as the selective use of fluconazole and antifungal rotation policies implemented in hospitals may have contributed to preventing resistance development.

Several meta-analyses reported voriconazole resistance in *C. albicans* strains to be 3.1% and 2.98% [63,65]. A study including 4226 strains from Spain found voriconazole resistance in 5% of the strains [70]. A meta-analysis from Iran reported voriconazole resistance in *C. albicans* strains to vary between 1.4% and 6.52% [6]. A multicenter study conducted in Europe determined that all *C. albicans* strains were susceptible to voriconazole [51]. A study conducted by the ARTEMIS DISK Antifungal Surveillance Program (2001–2005) and involving 124 centers worldwide reported the resistance rate of C. *albicans* to voriconazole as 1.2% [71]. In our study, the average voriconazole resistance was 4.37%. The voriconazole resistance rate obtained in our study agrees with the rates reported for European countries and Iran, supporting the global low resistance trend. This shows that the general susceptibility of *C. albicans* to voriconazole is preserved, and regional data should be considered in treatment approaches.

In Pakistan, dose-dependent itraconazole resistance in *C. albicans* strains was reported to be 28.9% [72]. Studies from Iran reported itraconazole resistance rates to vary between 4.8% and 16.65% [6,73,74]. Our study determined itraconazole resistance to be 4.42%. The wide variability in the rates reported for itraconazole resistance in different countries may be due to both the antifungal susceptibility detection methods used and different interpretations of resistance breakpoints. The low resistance rate obtained in our study may be associated with the use of standardized methods and careful determination of epidemiological boundaries.

Echinocandins are first-line antifungal drugs against azole-resistant *Candida* species due to their high efficacy. A meta-analysis study reported caspofungin and anidulafungin resistance to be 4.53% and 1.79%, respectively [6]. Another meta-analysis study indicated caspofungin and anidulafungin resistance as 3.96% and 1.61%, respectively [63]. A meta-analysis including data from South Africa and Cameroon reported no caspofungin-resistant strains, while anidulafungin resistance was indicated as 1.9% only in South Africa [66]. A study from Türkiye found all *C. albicans* strains to be susceptible to caspofungin [43]. A study including 188 *C. albicans* strains from Europe reported the resistance rate to caspofungin as 1% [64]. The present meta-analysis determined the average resistance to caspofungin, anidulafungin, and micafungin to be 2.94%, 1.05%, and 1.95%, respectively. Our data agree with the literature, showing that high efficacy against echinocandins continues.

Er et al. (2021) reported that all *C. albicans* strains included in their study conducted in the Aegean region of Türkiye were susceptible to posaconazole [49], while Carrillo-Muñoz et al. (2010) reported the resistance rate to posaconazole to be 4.06% [75]. Our study determined the posaconazole resistance of *C. albicans* strains to be 2.91% on average, and high heterogeneity was observed between studies. The reason for the heterogeneity was due to differences between geographical regions. Differences in species and genotypes of regionally dominant *Candida* strains may cause discrepancies in antifungal resistance patterns. Furthermore, prophylactic or therapeutic use of posaconazole in immunosuppressed patient groups with hematological malignancies may increase the selection pressure against the drug and pave the way for resistance development.

Flucytosine-5 has a broad antifungal spectrum against common pathogenic yeasts [76]. Barchiesi et al. (2000) reported that flucytosine-5 resistance in *C. albicans* ranged from 0% to 3% [77]. Kılbaş et al. (2017) indicated the average resistance rate for flucytosine-5 as 0.7 ± 1.3% in their meta-analysis study [9]. Different studies from Türkiye [24,29,52] found flucytosine-5 resistance to range from 0% to 3.57%. This meta-analysis determined flucytosine-5 resistance to be 0.29% on average. Flucytosine-5 resistance did not increase significantly and maintained its effectiveness in the studies reported in the literature from 2000 to the present and in the studies included in the current meta-analysis.

Significant heterogeneity was found for azole antifungal drugs, such as fluconazole (I^2^ = 88.73%) and itraconazole (I^2^ = 90.89%). This high heterogeneity may be due to differences in diagnostic methods, regional antifungal use, or study design, which may limit the generalizability of the pooled estimates. The lower heterogeneity in studies on resistance to flucytosine (I^2^ = 0%) and amphotericin B (I^2^ = 34.67%) indicates more consistent resistance rates, supporting the reliability of these combined rates.

The present study has some limitations. There are significant differences between the antifungal susceptibility testing methods used in the included studies, which may have increased the heterogeneity in the studies by limiting the comparability of antifungal resistance rates. Likewise, the preferences of the antifungal susceptibility interpretation guide, the medical history of the patients from whom strains were isolated, and the variety in the types of clinical samples used may also have contributed to the heterogeneity in the findings. The imbalance in the number of studies and sample distribution between geographical regions may also limit the generalizability of the results. In particular, studies conducted with low samples in some regions may cause resistance rates to appear high or low.

## 5. Conclusions

Sustainability and effective operation of nationally established surveillance programs to regularly and systematically monitor antifungal resistance trends in Türkiye are crucial. This allows treatment guidelines to be reshaped in light of current data. Furthermore, identifying differences in antifungal use across hospitals and regions will contribute to more accurate structuring of empirical treatment algorithms at both the regional and national levels. Meta-regression analyses revealed that geographic regions contribute significantly to heterogeneity, suggesting that antifungal resistance rates are not distributed homogeneously across Türkiye and that regional differences are significant. These differences may be caused by multiple factors, including antifungal drug access policies, hospital infection control practices, differences in laboratory infrastructure, and the resistance phenotypes of dominant *Candida* strains. Equalizing regional representation within national surveillance systems and standardizing data will reduce heterogeneity in future meta-analysis studies and provide more reliable estimates. Additionally, new antifungal drug development efforts should focus on regional resistance profiles and new methods that can overcome the existing resistance mechanisms. Incorporating real-time surveillance data into drug design studies can improve the clinical efficacy of antifungals and enhance regional compliance.

## Figures and Tables

**Table 1:** Characteristics of studies included in the meta-analysis [14,15,16,17,18,19,20,21,22,23,24,25,26,27,28,29,30,31,32,33,34,35,36,37,38,39,40,41,42,43,44,45,46,47,48,49,50,51,52,53,54,55].

Studies	Data Year	Geographical Region	Clinical Sample	Identification	Antifungal Susceptibility Detection Method	Antifungal Susceptibility Testing Guide
Kocoglu et al., 2005 [14]	2004	Marmara	Blood, urine, wound swab, sputum, stool, fasting gastric juice, drain fluid	API ID 32C	ATB Fungus	Unidentified
Yenisehirli et al., 2007 [16]	2003–2007	Black Sea	Blood	GTT, CCF-T80, API 20C AUX	BM, E-test	CLSI
Tulumoglu et al., 2009 [17]	2006–2008	Central Anatolia	Blood, urine, wound swab, catheter	API ID 20C AUX	API ATB Fungal 3 kit	Unidentified
Saracli et al., 2009 [18]	2001–2006	Central Anatolia	Blood	API ID 32C	BM	CLSI
Altuncu et al., 2010 [19]	-	Marmara	Blood, urine, CSF, PF, ETA, pleural fluid	API ID32C	BM	CLSI
Kiraz et al., 2011 [20]	2003–2008	Central Anatolia	Blood, urine, sputum, catheter, wound swab, stool, skin swab	CC, API 20C AUX	E-Test	CLSI
Belet et al., 2011 [21]	2004–2008	Black Sea	Blood, CSF, PF, pericardial fluid	API 20CAUX	E-Test	CLSI
Aydin et al., 2011 [22]	2005–2009	Black Sea	Blood	CHROMagar, API ID 32C	Disk diffusion, E-Test	CLSI
Ozbek et al., 2012 [23]	2009–2010	Southeastern Anatolia	Blood, urine, PF, sputum	VITEK^®^ 2	VITEK^®^ 2	Unidentified
Calıskan et al., 2013 [24]	2009–2012	Central Anatolia	Blood	VITEK^®^ 2	VITEK^®^ 2	Unidentified
Eksi et al., 2013 [25]	2008–2009	Southeastern Anatolia	Blood	API ID 32C	BM	CLSI
Cetin et al., 2014 [26]	2012–2013	Central Anatolia	Blood	VITEK^®^ 2	VITEK^®^ 2	CLSI
Yigit and Aktas, 2014 [27]	2011–2013	Eastern Anatolia	Blood	API 20 C AUX	BM	CLSI
Mutlu Sariguzel et al., 2015 [28]	2012–2014	Central Anatolia	Blood	VITEK^®^ 2	VITEK^®^ 2	CLSI
Hazirolan et al., 2015 [29]	2013–2014	Central Anatolia	Blood, urine, TA, wound swab, sputum, PF, catheter, abscess	VITEK^®^ 2	VITEK^®^ 2	CLSI
Yenisehirli et al., 2015 [15]	2007–2012	Black Sea	VCE	API 20C AUX	E-Test	CLSI
Etiz et al., 2015 [30]	2013–2014	Mediterranean	Blood	VITEK^®^ 2	VITEK^®^ 2	CLSI
Pelit and Uzun, 2015 [31]	2014–2015	Marmara	Urine, blood, TA, abscess	VITEK^®^ 2	VITEK^®^ 2	Unidentified
Hanci et al., 2015 [32]	2011–2014	Aegean	Blood, urine	API ID32C	API ATB Fungus 3	CLSI and EUCAST
Cekin et al., 2015 [33]	2010–2011	Mediterranean	Blood, urine, sputum, abscess, catheter, CSF	VITEK^®^ 2	VITEK^®^ 2	Unidentified
Turk Dagi et al.,2016 [34]	2010–2013	Central Anatolia	VCE	GTT, CCF-T80, API ID 32C	BM	CLSI
Ozer et al., 2016 [35]	2012–2013	Mediterranean	Urine	VITEK^®^ 2	VITEK^®^ 2	CLSI
Kucukates et al., 2016 [36]	2013–2014	Marmara	Blood, urine, sputum, wound swab, ETA	CC, API ID 32C	CMP	Unidentified
Aydemir et al., 2017 [37]	2011–2015	Marmara	Blood, urine, TA, wound swab, CSF, catheter	VITEK^®^ 2	VITEK^®^ 2	CLSI
Savci and Yilmaz, 2017 [38]	2014–2016	Central Anatolia	Urine, VS, BAL, sputum, wound swab	VITEK^®^ 2	VITEK^®^ 2	CLSI
Sirin et al., 2017 [39]	2012–2015	Aegean	Blood	API ID 32C ve API ATB Fungus 3	API ATB Fungus 3	CLSI
Calgin et al., 2018 [40]	2015–2017	Black Sea	Blood, urine, sputum, wound swab	VITEK^®^ 2	VITEK^®^ 2	Unidentified
Altin et al., 2018 [41]	2011–2012	Central Anatolia	Urine, blood, BAL, wound swab	VITEK^®^ 2	VITEK^®^ 2	Unidentified
Arikan-Akdagli et al., 2019 [42]	1997–2017	-	Blood	GTT, API ID 32C, API 20C AUX	BM	CLSI
Dogan et al., 2020 [43]	2015–2018	-	Blood	MALDI-TOF/MS	BM	CLSI
Beder et al., 2020 [44]	2014–2018	Central Anatolia	VCE	VITEK^®^ 2	VITEK^®^ 2	CLSI
Mete et al., 2020 [45]	2004–2008, 2013–2017	Marmara	VCE	API ID32	E-Test	EUCAST
Yakut et al., 2021 [46]	2013–2019	Marmara	VCE	API ID32 C, MALDI TOF MS	CMP	CLSI
Yilmaz-Ciftdogan et al., 2021 [47]	2012–2018	Aegean	Blood	API ID 32C	E-Test	CLSI
Dikmen et al., 2021 [48]	-	Mediterranean	Mouth swab	PCR	NA	CLSI and EUCAST
Er et al., 2021 [49]	2017–2019	Aegean	Blood	API ID 32C, MALDI-TOF MS	E-Test	EUCAST
Sanli et al., 2021 [50]	2015–2019	Marmara	VCE	PHOENİX M50 (BD USA	VITEK^®^ 2	CLSI
Alci et al., 2022 [51]	2017–2021	Marmara	Blood, urine, wound swab, catheter, VS, BAL and VCE	MALDI-TOF MS, VITEK 2	VITEK^®^ 2	EUCAST
Aydogan et al., 2022 [52]	2020–2022	Central Anatolia	Blood	VITEK 2	VITEK^®^ 2	EUCAST
Karvar and Delialioglu, 2023 [53]	2020	Mediterranean	Blood	VITEK 2	BM	EUCAST
Tosun and Kirisci et al., 2024 [54]	2022–2024	Mediterranean	Blood	BD Phoenix M50	BM	EUCAST
Kalkanli et al., 2024 [55]	2019–2020	Southeast Anatolia	Blood, urine, wound swab, VS, catheter, and VCE	MALDI-TOF MS	E-Test	EUCAST

TA: tracheal aspirates, CSF: cerebrospinal fluid, VS: vaginal swab, BAL: bronchoalveolar lavage, VCE: various clinical examples, ETA: endotracheal aspirate, PF: peritoneal fluid, GTT: Germ Tube Test, CCF-T80: corn flour and tween-80-containing medium, CC: CHROMagar *Candida*, MALDI-TOF MS: Matrix-Assisted Laser Desorption/Ionization Time-of-Flight Mass Spectrometry, BM: broth microdilution, CMP: colorimetric microdilution panel.

**Table 2:** Antifungal resistance status of the studies included in the meta-analysis [14,15,16,17,18,19,20,21,22,23,24,25,26,27,28,29,30,31,32,33,34,35,36,37,38,39,40,41,42,43,44,45,46,47,48,49,50,51,52,53,54,55].

Antifungal Agent	Number of Studies Reporting	Sample Size	Number of Resistant Isolates	Resistance Rate (%)	95% CI
Amp B	41	4718	117	2.10%	2.04–2.92%
5-FC	25	2423	16	0.29%	0.34–0.98%
FLC	41	5523	248	4.45%	3.94–5.04%
VRC	38	4144	181	4.37%	3.75–4.99%
ITZ	16	2894	128	4.42%	3.67–5.17%
CAS	19	2382	70	2.94%	2.26–3.62%
MFG	12	2509	49	1.95%	1.41–2.49%
POS	9	1546	45	2.91%	2.07–3.75%
ANF	8	571	6	1.05%	0.21–1.89%

5-FC: flucytosine-5, Amp B: amphotericin B, CI: confidence interval, FLC: fluconazole, VRC: voriconazole, ITZ: itraconazole, CAS: caspofungin, MFG: mikagunfin, POS: posaconazole, ANF: anidulafungin.

**Figure A1:**
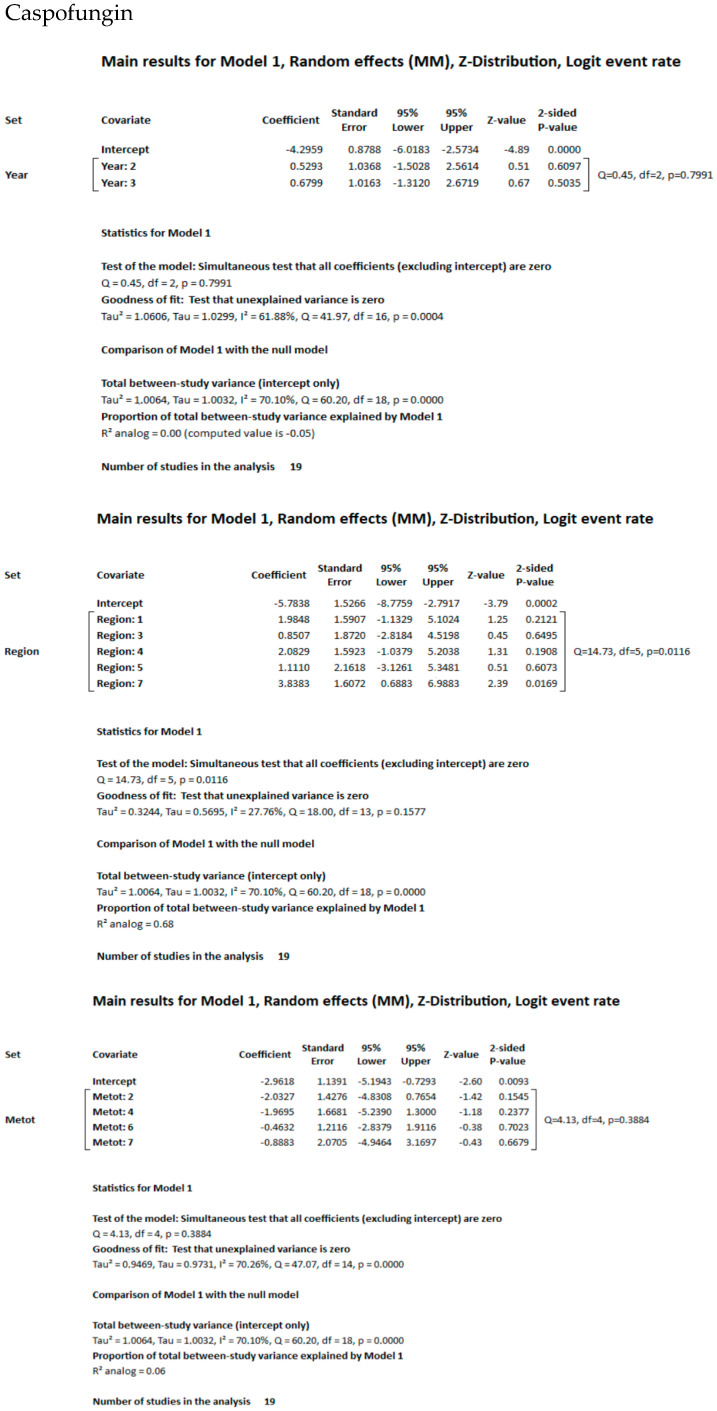

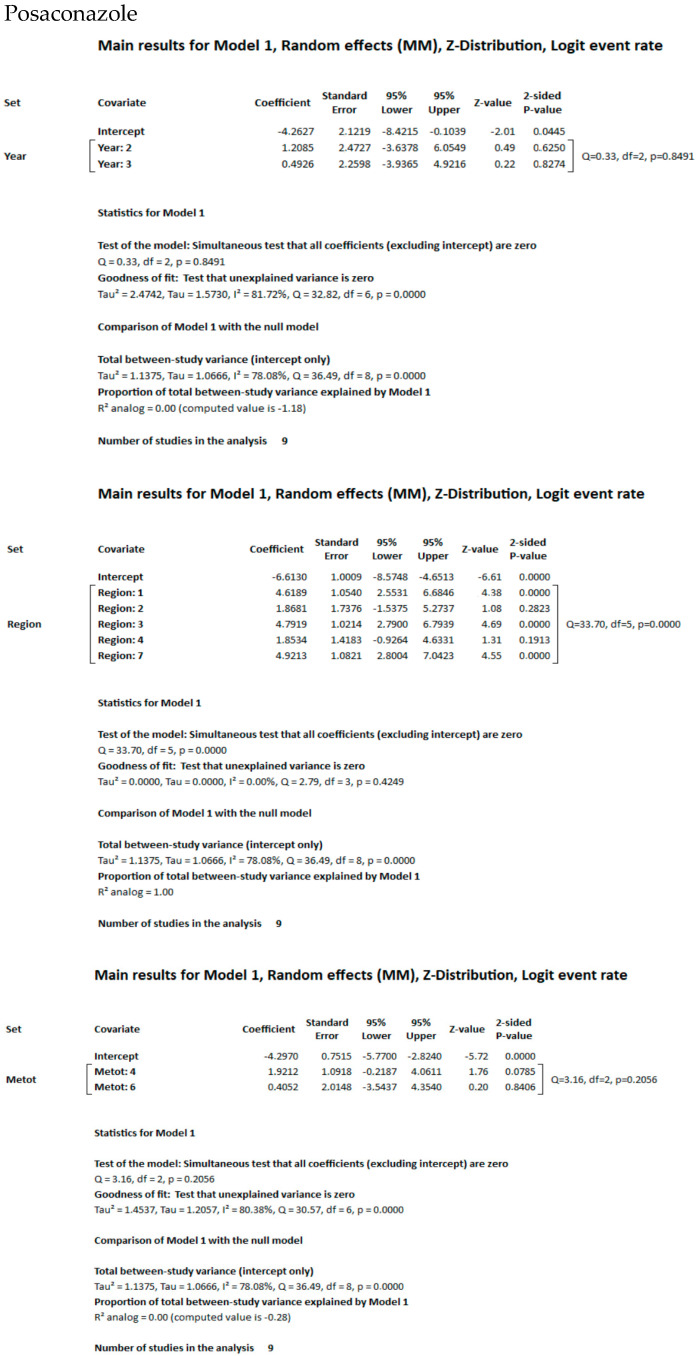

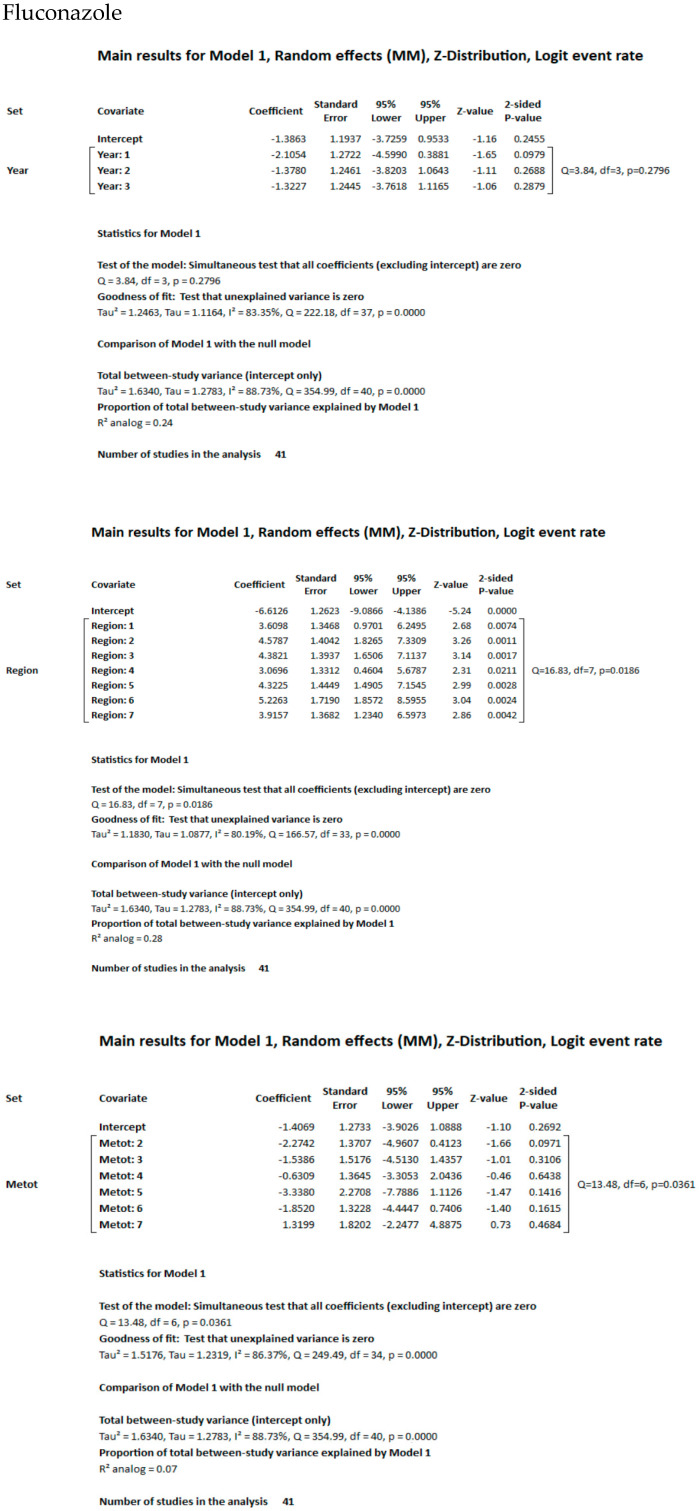

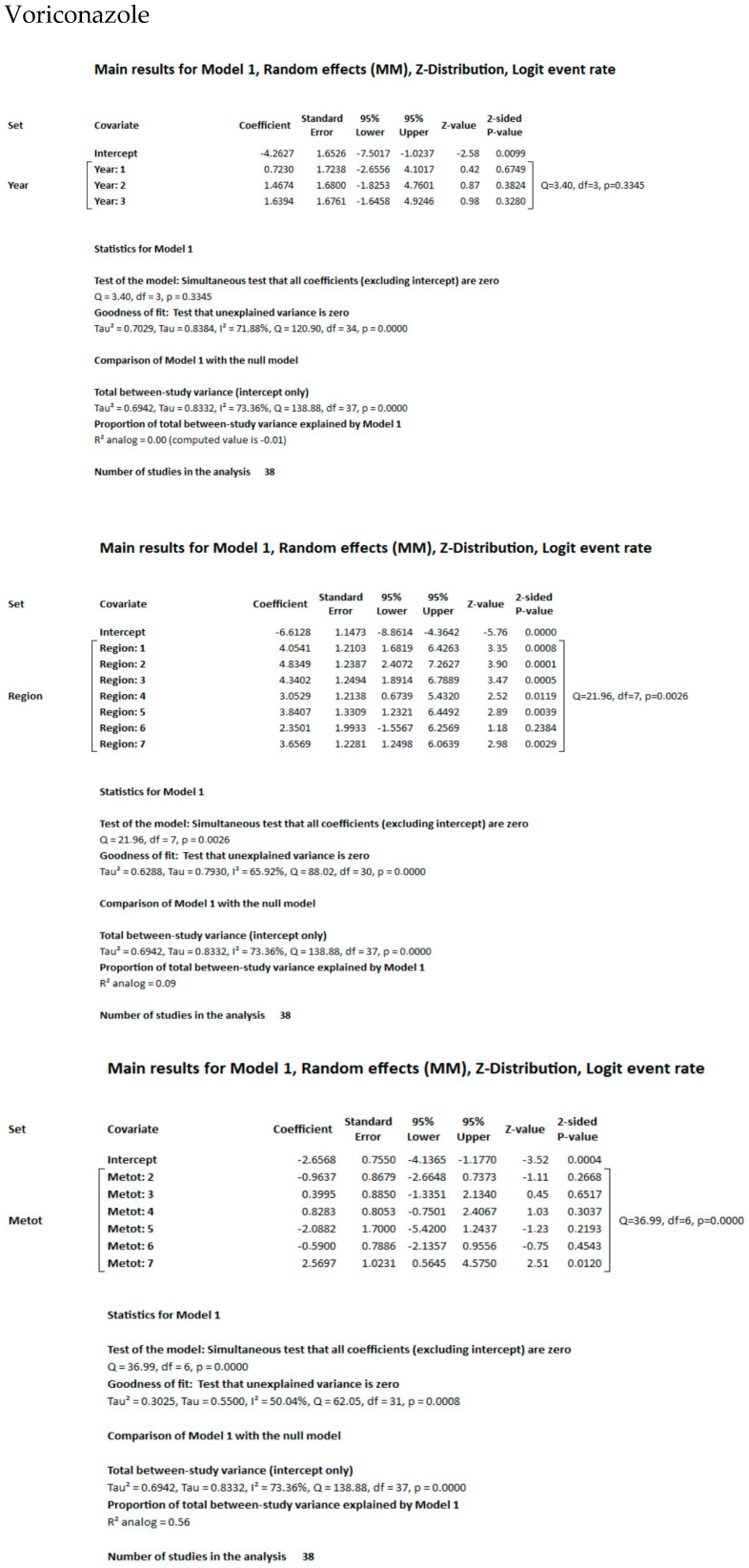
Meta-regression analysis of factors affecting antifungal resistance heterogeneity.

## Data Availability

The original contributions presented in this study are included in the article. Further inquiries can be directed to the corresponding author.
